# Concordance of Chest CT and Nucleic Acid Testing in Diagnosing Coronavirus Disease Outside its District of Origin (Wuhan, China)

**DOI:** 10.6061/clinics/2020/e1910

**Published:** 2020-08-14

**Authors:** Li-Qin Yang, Jin-Ming Cao, Tian-Wu Chen, Jian-Qiong Yang, Qi-Wen Mu

**Affiliations:** I Sichuan Key Laboratory of Medical Imaging, and Department of Radiology, Affiliated Hospital of North Sichuan Medical College, Nanchong, Sichuan, China.; II Department of Radiology, Nanchong Central Hospital/Second School of Clinical Medicine, North Sichuan Medical College, Nanchong, Sichuan, China.

**Keywords:** Coronavirus, COVID-19, Computed Tomography, Nucleic Acid Testing

## Abstract

**OBJECTIVES:**

This study aimed to determine the concordance between CT and nucleic acid testing in diagnosing coronavirus disease (COVID-19) outside its district of origin (Wuhan, China).

**METHODS:**

Twenty-three consecutive patients with COVID-19, confirmed by nucleic acid testing, were enrolled from two designated hospitals outside the district of disease origin. We collected clinical, laboratory, and CT data and assessed the concordance between CT manifestations and nucleic acid test results by comparing the percentage of patients with and without abnormal CT findings. Furthermore, using Chi-square tests, we analyzed the differences in CT manifestations between patients with and without an exposure history or symptoms.

**RESULTS:**

Multiple ground-glass opacities (GGOs), with or without consolidation, were observed on the initial CT scans of 19 patients (82.6%), whereas the remaining 4 (17.4%) showed no CT abnormalities, indicating that the initial chest CT findings were not entirely concordant with the nucleic acid test results in diagnosing COVID-19. Among the latter 4 patients, we observed multiple GGOs with and without consolidation in 2 patients on the follow-up chest CT scans taken on days 7 and 14 after admission, respectively. The remaining 2 patients showed no abnormalities on the follow-up CT scans. Furthermore, abnormal CT findings were found more frequently in patients who had been exposed to COVID-19 in its district of origin than in those who had not been exposed and in symptomatic patients than in asymptomatic patients (all *p*<0.05).

**CONCLUSIONS:**

Patients with positive results on nucleic acid testing may or may not have the abnormal CT manifestations that are frequently found in symptomatic patients with a history of exposure to the district of COVID-19 origin.

## INTRODUCTION

In December 2019, an outbreak of coronavirus disease (COVID-19) that originated in Wuhan City, Hubei Province (China), rapidly spread across the country and, in the following months, posed an enormous challenge to China’s public health (1). Since then, this disease has spread to many countries across the world, and it was declared a pandemic in March 2020 by the World Health Organization. Cases of COVID-19 are no longer limited to Mainland China, and infections have been reported in over 210 countries in the world, including South Korea, Italy, Japan, Iran, and Singapore (2).

A recent report revealed that the severe acute respiratory syndrome coronavirus 2 (SARS-CoV-2) could be transmitted from person to person mainly through infected droplets and close contact. It also stated that patients could potentially transmit the virus to other persons even in the incubation period of the disease (3). In addition, the mechanisms of COVID-19 transmission to patients outside the district of its origin include infection during a stay in the district of origin of COVID-19 (*i.e.* imported infection) and infection following close contact with individuals recently exposed to the virus in this district (*i.e.*, second-generation infection). The incubation period for COVID-19 is generally 1-14 days, however, symptoms may appear after 3-7 days, and the main clinical symptoms are fever, dry cough, and fatigue; a few patients have also reported symptoms such as runny nose, nasal congestion, and diarrhea. Acute respiratory distress syndrome, metabolic acidosis that is difficult to correct, septic shock, and coagulation dysfunction are the common complications in severe cases (4).

In accordance with the guidelines for the Diagnosis and Treatment of Pneumonitis Caused by COVID-19 (trial version 7), early diagnosis and isolation is essential to avoid continuously transmissive infection and to ensure early effective treatment (5). At present, viral nucleic acid detection using real-time polymerase chain reaction (RT-PCR) and chest CT examinations help in the diagnosis of COVID-19. According to related reports, the positive results obtained on nucleic acid testing, as a critical indicator of isolation or hospitalization in time, must be confirmed by RT-PCR of throat swabs, lower respiratory tract secretions, or sputum (6). As a routine imaging tool for pneumonia diagnosis, chest CT scan is relatively easy to perform and can provide a rapid diagnosis (7,8). As previously reported (9), some patients with confirmed disease outside the district of COVID-19 origin (Wuhan, China) might present with normal CT images during the incubation period or even in early stages of the disease, suggesting that the CT manifestations may not be consistent with RT-PCR results in the diagnosis of this disease. To the best of our knowledge, there are no reports focusing on the concordance between CT and nucleic acid testing in the diagnosis of COVID-19 among patients outside Wuhan, China. Thus, the purpose of our study was to investigate the concordance of initial CT manifestations and nucleic acid testing in diagnosing COVID-19 during the incubation period or in the early stage of the disease among patients outside the district of COVID-19 origin, aiming to help clinicians formulate more accurate and effective prevention and treatment measures.

## MATERIALS AND METHODS

### Patients

This retrospective study was approved by the Medical Ethics Committee of Affiliated Hospital of North Sichuan Medical College (approval number, 2020ER007-1) and was performed in accordance with the tenets of the Declaration of Helsinki. Informed consent was obtained from each patient.

From January 21 to February 23, 2020, we enrolled a total of 23 consecutive COVID-19 patients from two designated hospitals outside the district of origin (Wuhan, China) of this disease according to the following inclusion criteria: (1) positive results for SARS-CoV-2 via RT-PCR and (2) had undergone CT examinations after admission. The exclusion criteria were as follows: (1) lack of CT examination, or (2) likelihood of other primary diseases, particularly lung diseases such as fibrosis or emphysema. The cohort comprised 13 male (56.5%) and 10 female (43.5%) patients, and the mean age was 45.78±15.48 years (age range, 10 to 77 years). All patients were found to be positive for SARS-CoV-2, as assessed via RT-PCR on admission. In addition to age and sex, data on exposure history, chest CT findings, and clinical characteristics of these patients were collected (Table 1).

All patients were divided into two groups according to the manifestations observed on chest CT during admission. Nineteen patients with abnormal findings on the initial chest CT images were placed in Group A, and they were found to have multiple ground-glass opacities (GGOs) with/without consolidation or fibrosis. The remaining four patients, who had normal CT findings, formed the second group (Group B). In Group A, there were 12 male (63.2%) and 7 female (36.8%) patients, and the mean age was 47.74±13.12 years (age range, 24 to 77 years). In Group B, there was 1 male patient (25%) and 3 female patients (75%), and their mean age was 36.5±24.2 years (age range, 10 to 68 years).

According to the exposure history, patients in each group were divided into two subgroups: those with imported infection and those with second-generation infection. On the basis of the presentation of symptoms, the patients in each group were divided into symptomatic and asymptomatic subgroups. All patients with confirmed COVID-19 had been treated in isolation at hospitals and had received anti-viral therapy and other symptomatic supportive treatment depending on the severity of the disease. They underwent their first CT examinations and RT-PCR testing on the day of hospitalization and underwent regular follow-up CT scans and RT-PCR tests to observe the therapeutic effects during the treatments.

### CT image acquisitions

Non-contrast enhanced thoracic CT scans were performed in 17 patients with a 16-section multidetector row CT (MDCT) system (uCT 510, United Imaging, Shanghai, China) and in 6 patients with a 128-section MDCT system (SOMATOM Definition Flash, Siemens Healthcare systems, Germany) in the two designated hospitals. Scanning parameters for the uCT 510 scanner were as follows: Tube voltage=120 KV, tube current=200 mA (automatic exposure control employed), rotation time = 0.35s, pitch=1.5 mm, detector collimation=0.625 mm, and slice thickness/reconstruction thickness=5 mm/1 mm. The scanning parameters for SOMATOM Definition Flash scanner were similar to those for the 16-MDCT scanner except for the tube current (250 mA) and detector collimation (0.6 mm). Following the usual methodology for chest CT, all scans were performed during end-inspiration with the patients in the supine position. Data from two CT scanners were transferred to their respective image processing workstations (SOMATOM Definition Flash, Siemens Healthcare systems, Germany). Window settings included mediastinum window (width, 350 HU; level, 40 HU) and lung window (width, 1500 HU; level, −700 HU).

### CT data analysis

All CT data were reviewed by two experienced radiologists (readers with 7 and 9 years of experience in CT study), and a consensus was reached. Any disagreements in interpretation were adjudicated by the senior radiologist (the corresponding author, with 22 years of experience), and a final decision was reached. All images were viewed in both lung window and mediastinum window settings.

According to expert consensus (10), the CT findings for each patient were assessed on the basis of the presence of the following radiologic features: (1) no detectable abnormality, (2) subpleural pure GGO, and (3) consolidation. Another abnormality noted was lung fibrosis. It has been noted that GGO is considered a basic radiologic finding of COVID-19 on CT scans and appears as an area of hazy opacity that does not hide the underlying pulmonary vessels or bronchial structures. GGOs do not have solid components; therefore, when the underlying vessels are obscured in areas of increased density, the lesions are considered as consolidations.

### Statistical analysis

All data were statistically analyzed using IBM SPSS statistics software (version 22.0). Quantitative data are expressed as mean±standard deviation. Counting data are expressed as a percentage of the total. The concordance between CT manifestations and results of nucleic acid testing was assessed by comparison of percentages of patients with and without abnormal CT findings, and the Chi-square test was used to analyze the difference in CT manifestations between patients with different exposure histories or between patients with and without symptoms. All analyses were considered significant at *p*<0.05.

## RESULTS

### Concordance between CT manifestations and positive results of nucleic acid testing

**Figure 1:**
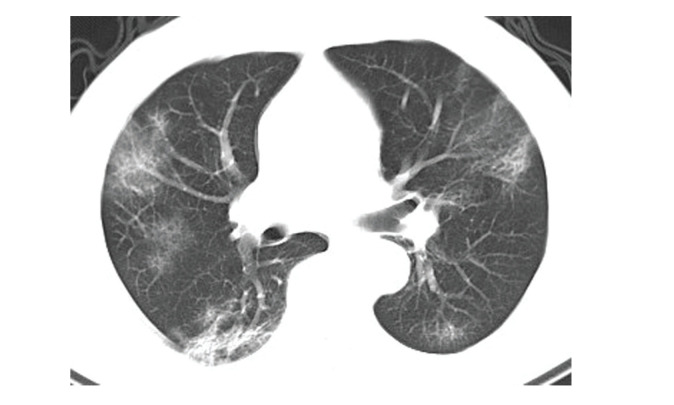
In a 60-year-old male patient with a history of exposure to the district of origin of COVID-19, the findings on the initial CT at admission are subpleural multiple GGOs in both lungs. COVID-19, coronavirus disease; GGOs, ground-glass opacities.

In our study cohort, the initial chest CT showed abnormal presentations in 19/23 (82.6%) patients (Figure 1), who were enrolled in Group A. The CT scans of 8 of these patients (42.1%) showed pure GGOs, those of a further 8 (42.1%) presented GGOs with consolidation, and those of the remaining 3 (15.8%) showed fibrosis; these findings were consistent with positive results on nucleic acid testing. The remaining 4 (17.4%) patients, who did not have abnormal CT findings but had positive results on nucleic acid testing, were enrolled in Group B. Concordance between the abnormal CT manifestations and positive results of nucleic acid testing could be observed in most patients, but in a small minority, normal CT findings and positive results of nucleic acid testing were observed. These results are shown in Table 1.

**Figure 2:**
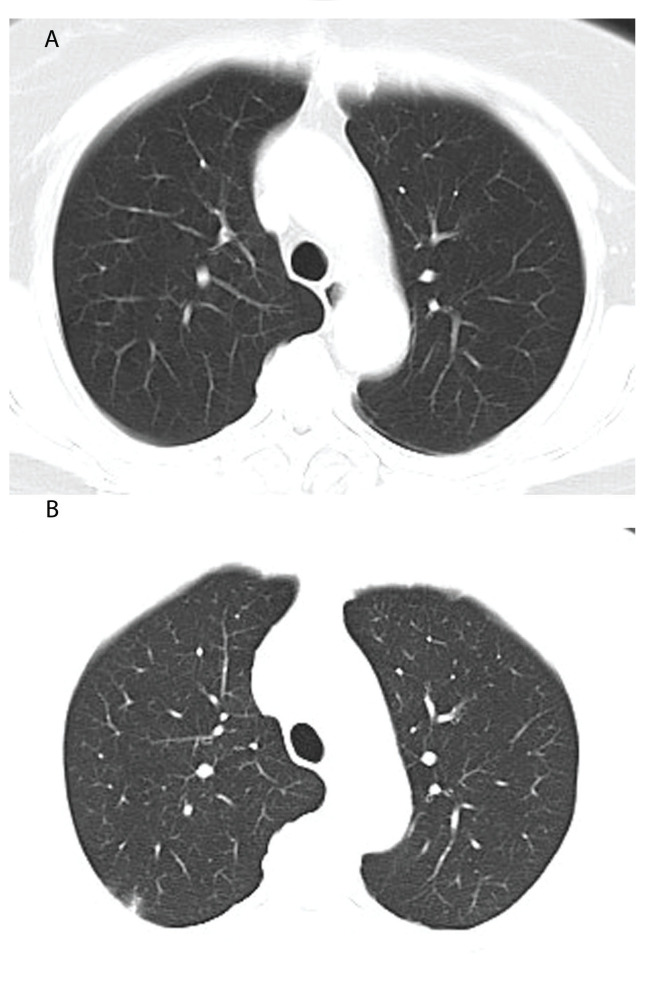
In a 68-year-old female with a history of close contact with patients who were recently exposed to the district of coronavirus disease origin (*i.e.* second-generation infection), the axial non-contrast CT scan (A) shows normal manifestations, and day 14 follow-up CT scan (B) shows subpleural mixed GGOs and fibrosis. GGOs, ground-glass opacities.

Of the 4 patients with initial normal CT findings, we observed multiple GGOs with and without consolidation in 2 patients on the follow-up chest CT scans taken on days 7 and 14 after admission, respectively (Figure 2). The remaining 2 patients, one aged 10 years and the other 29 years, had no abnormalities on the follow-up CT examinations.

### CT manifestations based on history of exposure to the district of disease origin (Wuhan)

Of the 23 patients, 17 had a history of exposure to the district of COVID-19 origin (Wuhan, China) whereas 6 did not. In Group A, history of exposure to the district of COVID-19 origin (*i.e.* imported infection) was found in 16 (84.2%) patients; the remaining 3 (15.8%) had no history of exposure but had close contact with patients who did (*i.e.* second-generation infection). In group B, 1 (25%) patient had an imported infection, while 3 (75%) patients had second-generation infection. A history of exposure to the district of COVID-19 origin was more frequently seen in patients belonging to Group A than group B (*p=*0.04).

### CT manifestations on the basis of presentation of symptoms

In Group A, 18 (18/19, 94.7%) patients were symptomatic, whereas 1 (1/19, 5.3%) patient was asymptomatic (Table 1). In Group B, 1 (1/4, 25%) patient was symptomatic, but the other 3 (3/4, 75%) were asymptomatic. Statistical analyses showed that most of the patients in Group A manifested clinical symptoms, whereas most of patients in Group B did not (*p=*0.009). A history of exposure to the district of COVID-19 origin was more frequently seen in symptomatic patients than in asymptomatic patients (16/17 *vs.* 3/6, *p=*0.04).

## DISCUSSION AND CONCLUSION

The COVID-19 outbreak has attracted worldwide attention and is characterized by rapid transmission, atypical clinical symptoms, and a difficulty in detecting and assessing early stages of the disease or its incubation period (11). According to current diagnostic criteria, viral nucleic acid detection by RT-PCR remains the formative evaluation procedure and standard for the diagnosis of COVID-19. Additionally, chest CT manifestations assist in identification of pneumonia from viral infection and early prediction of severe complications (12,13).

As shown in our study, concordance between abnormal CT manifestations and positive results on nucleic acid testing could be found in most patients, with a small number having normal CT findings inconsistent with the positive results on nucleic acid testing. Most patients whose initial chest CT images showed multiple GGOs with/without consolidation had a history of exposure to the district of origin (Wuhan) of this disease. Normal initial chest CT findings, however, were more frequently found in patients with second-generation infection. Possible reasons for this inconsistency may be explained in several recent reports (14-16). Error-prone replication and recombination are commonly seen in RNA viruses, leading to the production of viral progenies with highly diverse genomes, thus leading to a reduction in pathogenicity and virulence. We hypothesize that SARS-CoV-2, as a novel RNA virus, may also have similar characteristics and that gene mutation or recombination could lead to the reduction of pathogenicity and virulence in patients with second-generation infections.

Moreover, we found that there was good consistency between the CT manifestations and clinical symptoms in patients with COVID-19, suggesting that CT findings could correctly reflect the lesions of this disease. Most patients with abnormal CT manifestations were symptomatic, whereas patients with normal CT manifestations were often asymptomatic. It should be emphasized that patients with asymptomatic infections (*i.e.* infectious disease during the incubation period) may also be a source for future, second-generation infections. These asymptomatic patients can be difficult to diagnose and isolate in time, and thus they tend to infect other people in a family, making it harder to prevent the spread of the disease (17,18). Among asymptomatic patients, COVID-19 can be diagnosed on the basis of viral nucleic acid detection tests using RT-PCR and on the basis of a history of exposure to the district of disease origin (Wuhan) or of close contact with patients recently exposed to this district. Limitations may exist in using CT for the early detection of asymptomatic patients with second-generation infections. However, nucleic acid testing could be essential for detecting infected patients, and follow-up CT scans might also be necessary to detect the possibility of slow-developing pneumonia.

Our study had two inevitable limitations. First, the sample size was relatively small, and there were very few patients in Group B (asymptomatic second-generation infections). Second, during this urgent period, there were no available lung tissue biopsies for investigation of the correlation between radiologic and histopathology findings.

In conclusion, the initial chest CT findings at admission were not entirely concordant with nucleic acid testing results in the diagnosis of COVID-19. Most patients with abnormal CT findings could have a history of exposure to the district of disease origin (Wuhan, China), while normal initial chest CT findings can be found more frequently in patients with second-generation infection. We hope that our findings will help in the detection of COVID-19 patients during the incubation period or at an early stage of the disease.

## AUTHOR CONTRIBUTIONS

Yang LQ, Cao JM, Yang JQ and Chen TW made significant contributions to the data acquisition, analysis and interpretation. Chen TW and Mu QW contributed to the concept and design. Yang LQ and Chen TW contributed to the manuscript writing. All of the authors read and approved the final version of the manuscript.

## Figures and Tables

**Table 1:** Summary of Patient Characteristics (n=23).

Clinical Characteristics		Patients (%)	
		Group A (n=19)	Group B (n=4)
Sex			
	Male	12 (63.2%)	1 (25%)
	Female	7 (36.8%)	3 (75%)
Age (years)		47.7±13.1	36.5±24.2
District of origin			
	Yes	16 (84.2%)	1 (25%)
	No	3 (15.8%)	3 (75%)
Clinical symptoms			
	Yes	18 (94.7%)	1 (25%)
	No	1 (5.3%)	3 (75%)
CT findings			
	GGO	8 (42.1%)	0
	Consolidation	8 (42.1%)	0
	Fibrosis	3 (15.8%)	0
	Normal finding	0	4 (17.4%)

Note: GGO=Ground-glass opacity.
